# Is Arctic medicine a distinct science? A Russian perspective

**DOI:** 10.3402/ijch.v72i0.21248

**Published:** 2013-05-09

**Authors:** Dimitrii G. Tikhonov

**Affiliations:** Yakut Scientific Centre of Complex Medical Problems, Siberian Branch of the Russian Academy of Medical Sciences, Sergelyakhskoe shosse, Yakutsk, Sakha Republic, Russian Federation 4677019. Email: aitri@mail.ru

Results of research conducted over the past 50 years in the Arctic region showed that the health of the population is influenced by many factors, including the physical environment, climate, genetics, health-related behaviours, and living conditions. Health care in the Far North also faces serious challenges, which could be improved if we have more knowledge of the risk factors, pathogenesis and clinical manifestations of human disease that may be unique to the region and its people. It is my intent to bring to the attention of our international colleagues the need to recognize a distinct branch of science called Arctic Medicine. In Russia, the establishment of Arctic Medicine was proposed as early as 1981 at a national conference in Krasnoyarsk.

There has been a long-standing interest in the effects of cold on the human body, not least among military strategists and battlefield personnel. It should be noted that Marshal Zhukov believed that the German invasion of the Soviet Union during the Second World War ultimately failed because of gross miscalculation in not providing adequate protection of their soldiers from the cold.

In the early 1950s, several northern researchers such as S.I. Slavin proposed to systematize and strengthen the organization of scientific research in the North. At the direction of the Council of Ministers of the USSR, the Presidium of the Academy of Sciences established a permanent Commission on the North in May 1954. In February 1960, the Presidium of the Supreme Soviet issued a decree *On improving the benefits to those working in the Far North and in areas equivalent to the Far North* in recognition of the massive population migration into the North which accompanied intensified exploitation of natural resources. In November 1967, Resolution No. 1029 of the Council of Ministers approved the *List of regions and areas equivalent to the Far North*. The official definition of “numerically small peoples” used in the USSR and Russian Federation has traditionally excluded the Sakha (Yakut) and Komi people, who have their own republics. A.P. Avtsyn called them great nations of the North. Their health conditions are similar to other indigenous people despite their large population size.

Within Russia, scientific research on acclimatization and adaptation to severe climatic conditions of the North was initiated in the 1950s, and was the focus of a conference of the USSR Academy of Medical Sciences in July 1957 in Irkutsk with the participation of scientists and physicians from Eastern Siberia and the Far East, and a meeting in November 1960 of the Standing Committee on the North of the USSR. This was paralleled internationally by the International Conference on Problems of Medicine and Health in the Arctic and Antarctica in Geneva in 1962, and later the first International Symposium of Circumpolar Health in 1967 in Fairbanks, Alaska, which came full circle in 2013 when the 15th congress returned to Fairbanks.

How is Arctic medicine defined? Arctic medicine is a branch of medical science that studies the features of the functioning of the human body and its diseases in the Arctic under extreme conditions of cold and photoperiod disruption in order to develop the most effective, practical and acceptable means of prevention, diagnosis and treatment. Arctic Medicine integrates the achievements of diverse fields of theoretical and applied medical sciences. This concept is further discussed and developed in my book *Arctic Medicine* (1).

Researchers are interested in answering the question: “How are diseases in the Arctic special?” In the 1980s, when I was a young doctor, one professor asked me: “How can stomach ulcer in Verkhoyansk be different from stomach ulcer in Moscow?” and immediately answered it himself: “There's no difference!” Such an attitude has been refuted by numerous medical scientists who live and work in the Far North, notably Professor A.A. Bezrodnih of Yakutsk State University (now the North-Eastern Federal University).

A noted researcher of biomedical problems of the North, A.P. Avtsyn of the USSR Academy of Medical Sciences, remarked in his 1985 book *Human Pathology in the North* that, while Arctic Medicine may be well known to Soviet doctors, it is not so among foreign researchers, who did not see it as having an independent theory and practice, unlike tropical medicine, for example. With some 800 million people in tropical Africa alone, compared to only 10 million in the Arctic, the comparison is hardly fair. The control of infectious diseases in the North is no less impressive than in tropical Africa. For example, in Yakutia, smallpox was eradicated in 1936, malaria in 1964, trachoma in 1967 and leprosy in the late 1970s.

It is undeniable that health care in the North is ineffective, which can be explained by the difficulties of training and recruitment of qualified staff knowledgeable about conditions in the North, affecting their ability to conduct prevention and treatment programs directed at the socially important diseases.

V.I. Hasnulin wrote in his 1998 book *Introduction to Polar Medicine* that many medical scientists, who live in the middle latitudes and have never lived in the North itself, claim that there is no particular problem for human health in high latitudes. They believe that there cannot be peculiarities in the functioning of homeostatic systems in the human body in the North, and extreme climate and geophysical factors have no influence on the development of a disease. The accumulated research of scholars such as A.P. Avtsyn, V.P. Kaznacheev, N.R. Deryapa, N.B. Vasilev, A.E. Panin, G.M. Danishevsky, etc. is often dismissed as eccentric or mere artefacts.

Plans for intensive industrial development in the North will create new conditions for the further development of Arctic Medicine. The recent reorganization of higher education and scientific research in Russia has led to the creation of new federal universities in 2010: the M.V. Lomonosov Northern Federal University and the M.K. Ammosov North-Eastern Federal University. Priority programs in Arctic medical research have been established in these institutions.

At the beginning of the 21st century, the disease profile of the population of the North has changed dramatically. There is a sharp increase in chronic diseases such as cardiovascular diseases and diabetes. Indeed, research conducted under the WHO registry program for acute myocardial infarction identified the population of Yakutsk as having an incidence of myocardial infarction that is among the highest in the world. Yakutsk has recorded some of the greatest temperature extremes in the world, from a low of −64.4°C in February 1891, to a high of +38.4°C in July 2011, a difference that could exceed 100°.

In conclusion, Arctic Medicine needs to be recognized as a distinct branch of medical science and promoted in education, research and health care delivery if the health of northern peoples is to improve and the quality of their health care is to be advanced and modernized.

*Dimitrii G. Tikhonov*
Yakut Scientific Centre of Complex Medical Problems
Siberian Branch of the Russian Academy of Medical Sciences
Sergelyakhskoe shosse, Yakutsk
Sakha Republic, Russian Federation 4677019
Email: aitri@mail.ru

